# Severe thrombocytopenia is associated with arterial thrombotic manifestations in antiphospholipid syndrome: a two-center cohort study

**DOI:** 10.3389/fimmu.2026.1927065

**Published:** 2026-08-26

**Authors:** Jie Kong, Yijing Su, Xinyun Zhu, Jingjing Zhao, Haojie Chen, Xiaoxiang Chen, Yingying Gao

**Affiliations:** 1 Department of Rheumatology and Immunology, Nantong First People’s Hospital, Southeast University, Nantong, Jiangsu, China; 2 Department of Rheumatology and Immunology, Affiliated Nantong Clinical College of Nantong University, Nantong First People’s Hospital, Nantong, Jiangsu, China; 3 Department of Rheumatology and Immunology, Renji Hospital Affiliated to Shanghai Jiao Tong University School of Medicine, Shanghai, China; 4 Department of Allergy and Immunology, Renji Hospital Affiliated to Shanghai Jiao Tong University School of Medicine, Shanghai, China

**Keywords:** antiphospholipid antibodies, antiphospholipid syndrome, arterial thrombosis, cerebral infarction, thrombocytopenia

## Abstract

**Objective:**

Thrombocytopenia in APS is often regarded primarily as a bleeding-related manifestation, but the relationship between platelet-count severity and thrombotic manifestations remains uncertain. We evaluated the association between severe thrombocytopenia and arterial thrombotic manifestations in patients with antiphospholipid syndrome (APS).

**Methods:**

In a two-center baseline cohort of 313 patients with APS, baseline platelet count (PLT) was categorized as PLT <50×10^9^/L, PLT 50–<100×10^9^/L, or PLT ≥100×10^9^/L. Associations with prevalent baseline thrombotic manifestations were assessed using multivariable logistic regression in 300 patients with primary APS or systemic lupus erythematosus-associated APS. In the Renji follow-up cohort (n=254), 48-month thrombotic events were evaluated using Kaplan–Meier curves and Cox models.

**Results:**

PLT <50×10^9^/L was associated with a higher frequency of arterial thrombosis (66.7%), particularly cerebral infarction (62.7%), whereas venous thrombosis was most frequent in the PLT 50–<100×10^9^/L group (68.4%). After multivariable adjustment, PLT <50×10^9^/L remained associated with a higher prevalence of arterial thrombosis (aOR 2.16, 95% CI 1.07–4.35, *P* = 0.032) and cerebral infarction (aOR 2.37, 95% CI 1.18–4.73, *P* = 0.015). During 48-month follow-up, PLT <50×10^9^/L was associated with increased risks of any thrombotic event (HR 3.28, 95% CI 1.54–6.97, *P* = 0.002), arterial thrombotic event (HR 3.95, 95% CI 1.39–11.21, *P* = 0.010), and cerebral infarction (HR 4.11, 95% CI 1.44–11.73, *P* = 0.008), but not venous-related events.

**Conclusion:**

Severe thrombocytopenia was associated with arterial thrombotic manifestations in APS, particularly cerebral infarction. PLT <50×10^9^/L may provide additional clinical risk information but may also reflect cumulative disease burden and overall APS severity.

## Introduction

Antiphospholipid syndrome (APS) is an acquired autoimmune thrombotic disorder mediated by persistent antiphospholipid antibodies (aPLs), and is characterized by arterial thrombosis, venous thrombosis, and pregnancy morbidity (1–3). In addition to these classical manifestations, several non-criteria features, including thrombocytopenia, hemolytic anemia, cardiac valve disease, microangiopathy, and neurological involvement, are increasingly recognized as clinically relevant components of the APS spectrum (4). The 2023 ACR/EULAR classification criteria incorporated hematological manifestations into the weighted clinical domains, underscoring the clinical relevance of thrombocytopenia in APS phenotype characterization (5).

Thrombocytopenia is one of the common hematological abnormalities in APS (6). It has traditionally been regarded as an immune thrombocytopenia-like manifestation or a bleeding-related concern, particularly when platelet counts (PLT) are markedly reduced, which may complicate anticoagulant or antiplatelet treatment decisions (7). However, APS-related thrombocytopenia does not necessarily indicate a low thrombotic risk (8, 9). Mechanistically, aPLs can activate platelets, endothelial cells, and monocytes, and may promote complement activation, neutrophil extracellular trap formation (NETosis), and thromboinflammatory responses (10–13). Persistent platelet activation and consumption may therefore contribute to reduced PLT, suggesting that thrombocytopenia in APS may reflect an activated immunothrombotic state rather than a purely hemorrhagic phenotype (14).

Previous studies have suggested that thrombocytopenia in APS is associated with higher adjusted Global Anti-Phospholipid Syndrome Score (aGAPSS), high-risk antiphospholipid antibody profiles, and increased risks of subsequent thrombotic events (15–17). However, most studies have defined thrombocytopenia using a single threshold of PLT <100×10^9^/L and treated it as a binary variable, without adequately distinguishing the clinical significance of different degrees of platelet reduction (7–9). Whether severe thrombocytopenia, particularly PLT <50×10^9^/L, is preferentially associated with arterial thrombosis, especially cerebral infarction, remains unclear.

We hypothesized that PLT <50×10^9^/L would be associated with a higher prevalence of arterial thrombotic manifestations at baseline and a greater risk of subsequent arterial thrombotic events, particularly cerebral infarction. To test this hypothesis, we included a two-center baseline APS cohort and stratified patients by baseline platelet count into PLT <50×10^9^/L, PLT 50–<100×10^9^/L, and PLT ≥100×10^9^/L groups. We compared clinical, laboratory, and antibody profiles across platelet strata and used multivariable logistic regression and Cox proportional hazards models to evaluate associations with prevalent baseline thrombotic manifestations and 48-month follow-up thrombotic events.

## Methods

### Study population

This two-center observational cohort study consecutively included patients with APS who underwent baseline assessment at Renji Hospital and Nantong First People’s Hospital between July 2017 and December 2025. Baseline PLT was defined as the first platelet count measured at the index admission before any initiation, escalation, or modification of glucocorticoid, immunosuppressive, biologic, or antithrombotic treatment during that hospitalization. Some patients had been receiving maintenance treatment before admission. Patients were stratified into the severe thrombocytopenia group (PLT <50×10^9^/L), moderate thrombocytopenia group (PLT 50–<100×10^9^/L), and reference group (PLT ≥100×10^9^/L). The threshold of PLT <50×10^9^/L was selected *a priori* as a clinically meaningful severity threshold rather than a data-derived cutoff or a previously validated APS-specific arterial-risk cutoff, because platelet counts below this level are commonly associated with increased bleeding-related precautions and may influence antithrombotic and immunosuppressive treatment decisions (18, 19). Eligible patients were required to fulfill the 2006 revised Sydney classification criteria for APS (1), have available baseline PLT and key clinical data, complete baseline aPL testing, and have sufficient information to determine baseline thrombotic manifestations and APS subtype. Patients with definite non-APS-related causes of thrombocytopenia or insufficient information to determine platelet strata or thrombotic status were excluded.

**Figure 1:**
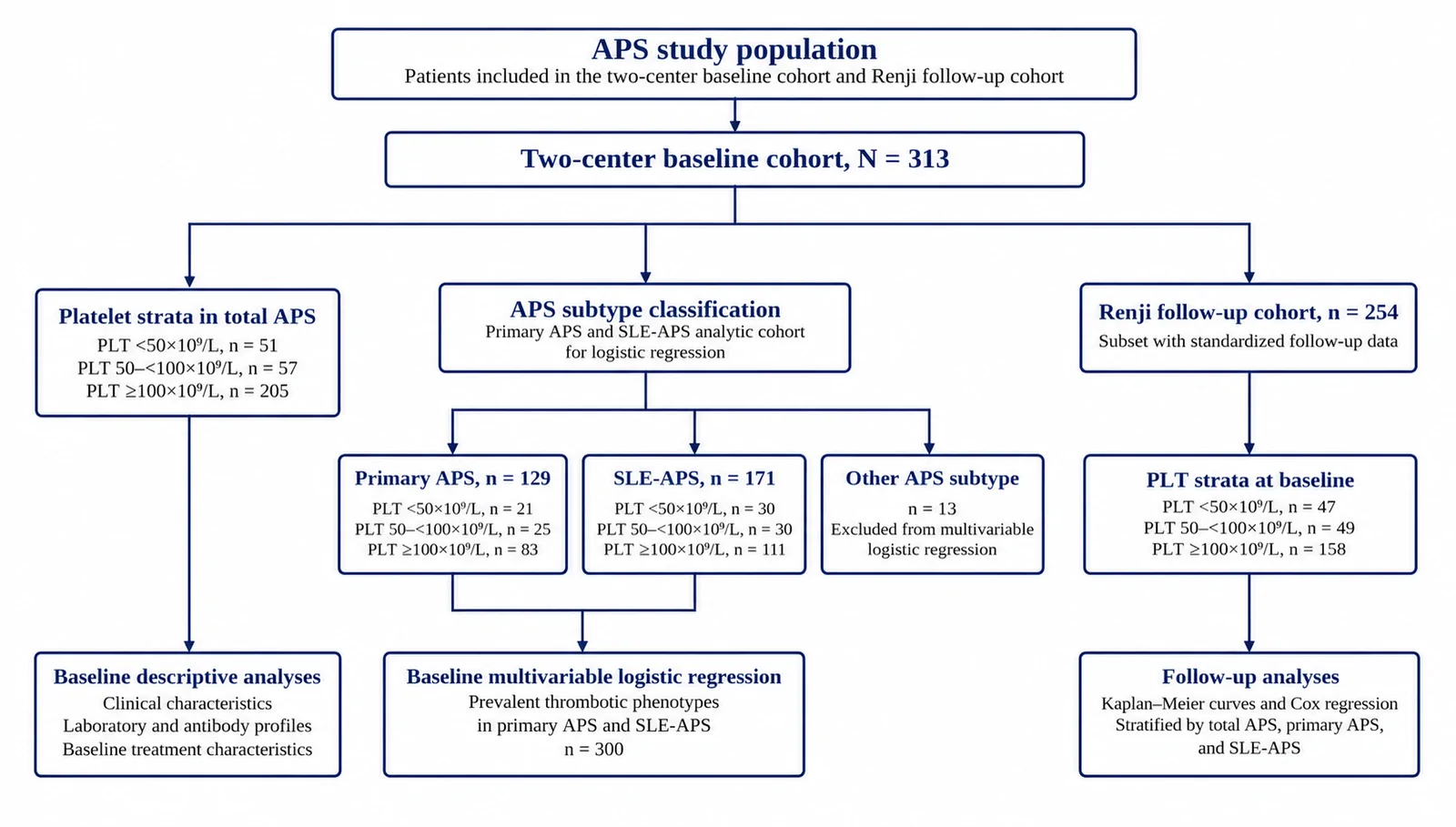
Flow diagram of the study population and analytic cohorts. A total of 313 patients with APS were included in the two-center baseline cohort and stratified according to the platelet count. Baseline descriptive analyses were performed in the total APS cohort. Multivariable logistic regression analyses were restricted to patients with primary APS or SLE-associated APS after exclusion of 13 patients with other APS subtypes. Follow-up analyses were performed in the Renji follow-up cohort of 254 patients with standardized longitudinal data.

A total of 313 patients were included in the two-center baseline cohort, including 254 from Renji Hospital and 59 from Nantong First People’s Hospital. Standardized longitudinal data were unavailable for the 59 Nantong patients; therefore, follow-up analyses were restricted to the 254-patient Renji cohort. According to APS subtype, patients were classified as primary APS, systemic lupus erythematosus-associated APS (SLE-associated APS), or other APS subtypes. The cohort was also reassessed according to the 2023 ACR/EULAR APS classification criteria using complete medical records. This study was approved by the ethics committees of the participating centers. Given the retrospective nature of the study and the use of anonymized data, the requirement for informed consent was waived. The study flow diagram and analytic cohorts are shown in **Figure 1**.

### Clinical and laboratory data collection

Baseline demographic, clinical, laboratory, antibody, and treatment data were collected from medical records. Demographic and general variables included age, sex, study center, disease duration, body mass index (BMI), APS subtype, traditional cardiovascular risk factors, aGAPSS, and Global Anti-Phospholipid Syndrome Score (GAPSS).

Baseline clinical manifestations included bleeding events, thrombotic events, obstetric morbidity, isolated immune thrombocytopenia (ITP), microangiopathy, cardiac valve disease, and autoimmune hemolytic anemia. Thrombotic events were classified as arterial thrombosis, cerebral infarction, myocardial infarction, visceral arterial thrombosis, venous thrombosis, deep venous thrombosis, pulmonary embolism, visceral venous thrombosis, or cranial venous sinus thrombosis. Cerebral infarction was defined as an arterial ischemic stroke confirmed by compatible neurological symptoms and objective neuroimaging evidence, including brain CT or MRI (20). Cranial venous sinus thrombosis was categorized as a venous-related thrombotic event. Visceral arterial thrombosis included aortic, mesenteric arterial, splenic arterial, renal arterial, and adrenal arterial thrombosis. Visceral venous thrombosis included portal venous, hepatic venous, splenic venous, mesenteric venous, and inferior vena cava thrombosis.

Laboratory variables included platelet count, hemoglobin, complement C3 and C4, erythrocyte sedimentation rate (ESR), and C-reactive protein (CRP). Antibody variables included anticardiolipin antibodies (aCL) IgA/IgM/IgG, anti-β2-glycoprotein I (anti-β2GPI) IgM/IgG antibodies, anti-phosphatidylserine/prothrombin antibodies (anti-PS/PT) IgM/IgG, anti-dsDNA antibodies, and anti-platelet antibodies. The autoantibody reference ranges were as follows: aCL IgA/IgM/IgG <12 U/mL, anti-β2GPI IgM/IgG cut-off index <1.0, anti-PS/PT IgM/IgG <30 units, and anti-dsDNA <77 IU/mL by ELISA. Lupus anticoagulant (LA) was detected using coagulation-based assays and was considered positive when the standardized LA ratio was >1.20 (21, 22).

Baseline treatment information included antiplatelet therapy, anticoagulation, warfarin, low-molecular-weight heparin, direct oral anticoagulants (DOACs), glucocorticoids, hydroxychloroquine, conventional immunosuppressants, anti-CD20 therapy, belimumab, and telitacicept. Baseline treatment variables represented therapies being received at admission or initiated during the index hospitalization and therefore did not necessarily indicate treatment-naïve status.

### Baseline thrombotic phenotypes and follow-up outcomes

Baseline thrombotic manifestations included any thrombosis, arterial thrombosis, cerebral infarction, and venous thrombosis. These outcomes represented prevalent manifestations documented at or before the index baseline assessment. Because platelet count and thrombotic history were assessed at the same baseline time point, the logistic regression analyses were cross-sectional and were designed to evaluate associations with prevalent thrombosis rather than temporal or causal relationships. Multivariable logistic regression was performed with PLT ≥100×10^9^/L as the reference group. Analyses were restricted to patients with primary APS or SLE-associated APS and complete covariate data (n=300), after excluding patients with other APS subtypes (n=13). Model 1 was adjusted for age, sex, and center; Model 2 was additionally adjusted for diabetes mellitus, smoking, and APS subtype; and Model 3 was further adjusted for aGAPSS. A sensitivity model additionally adjusted for disease duration was performed. Both PLT <50×10^9^/L and PLT 50–<100×10^9^/L were included in the models.

To evaluate whether cardiac valve disease or microvascular manifestations accounted for the observed associations, two additional sensitivity analyses were performed. First, extended multivariable logistic regression models simultaneously included both thrombocytopenia strata, cardiac valve disease, and microvascular disease, in addition to age, sex, study center, diabetes mellitus, smoking, APS subtype, and aGAPSS. Second, the primary three-stratum models were repeated after excluding all patients with cardiac valve disease or microvascular disease. To evaluate potential confounding by a high-risk serological profile, alternative sensitivity models replaced aGAPSS with triple-positive aPL profile. Because aGAPSS contains both serological and cardiovascular components, hypertension and hyperlipidemia were included as separate covariates in these models. Baseline logistic models were adjusted for age, sex, study center, hypertension, hyperlipidemia, diabetes mellitus, smoking, APS subtype, triple-positive aPL profile, and both platelet-count strata. Cox sensitivity models were adjusted for age, sex, APS subtype, triple-positive aPL profile, and both platelet-count strata.

Follow-up analyses were performed in the Renji follow-up cohort. Follow-up started from the baseline assessment date and was truncated at 48 months. The primary follow-up outcomes combined first-ever and recurrent thrombotic events occurring after baseline, including any thrombotic event, arterial thrombotic event, cerebral infarction, and venous-related thrombotic event. Events were confirmed by clinical manifestations and imaging or other objective examinations. Patients were censored at the outcome of interest, the last available follow-up visit, or 48 months, whichever occurred first. Follow-up events were additionally classified according to the corresponding baseline thrombotic history. An incident event was defined as the first occurrence of the corresponding thrombotic category in a patient without that category at baseline, whereas a recurrent event was defined as a new event occurring during follow-up in a patient with a history of the corresponding thrombotic category at baseline. Because category-specific event counts were limited, incident and recurrent events were primarily summarized descriptively. An exploratory analysis of recurrent any thrombosis was restricted to patients with any thrombosis at baseline. A restricted Kaplan–Meier analysis was additionally performed among patients without arterial thrombosis at baseline to evaluate first-ever arterial events; because only nine events occurred, adjusted Cox modeling was not performed. Mortality and catastrophic antiphospholipid syndrome (CAPS) were also reviewed during follow-up and summarized descriptively because of the very small number of events.

Cox proportional hazards models were used to evaluate the association between platelet strata and follow-up thrombotic events, with PLT ≥100×10^9^/L as the reference group. The total APS model was adjusted for age, sex, and APS subtype, whereas subgroup models for primary APS and SLE-associated APS were adjusted for age and sex. An extended Cox sensitivity model for the total APS cohort additionally adjusted for baseline arterial thrombosis, baseline venous thrombosis, antiplatelet therapy, anticoagulation, hydroxychloroquine, glucocorticoid use, and anti-CD20 therapy. Platelet strata were treated as fixed baseline exposures throughout follow-up, and longitudinal changes in platelet counts, treatment response, and time-dependent treatment modifications were not incorporated into the survival models. Treatment variables were entered as baseline covariates; because treatment selection was not randomized and may have been influenced by thrombocytopenia severity, previous thrombosis, or other APS manifestations, confounding by indication could not be fully eliminated.

### Statistical analysis

Continuous variables are presented as medians with interquartile ranges, and categorical variables as numbers and percentages. Comparisons among the three platelet strata were performed using the Kruskal–Wallis test for continuous variables and the *χ^2^* test or Fisher’s exact test for categorical variables, as appropriate. For variables with significant overall differences, pairwise comparisons were performed with Holm correction for multiple testing. Significant pairwise differences are indicated by superscript letters in the tables; within the same row, groups sharing a letter were not significantly different at *P* < 0.05.

Multivariable logistic and Cox regression models were constructed as described above. Logistic regression results are reported as adjusted odds ratios (aORs) with 95% confidence intervals (CIs), and Cox regression results as hazard ratios (HRs) with 95% CIs. Kaplan–Meier curves were used to compare event-free survival across platelet strata, with differences assessed using the log-rank test. The proportional hazards assumption was assessed using Schoenfeld residuals, and the results for the primary Cox models are shown in **Supplementary Table 1**. For recurrent any thrombosis, time to the first new arterial or venous thrombotic event was analyzed among patients with any thrombosis at baseline. Given the limited number of recurrent events, this analysis was considered exploratory, and the primary recurrent-event Cox model was adjusted for age, sex, and APS subtype to reduce the risk of model overfitting.

Sensitivity analyses are reported in the **Supplementary Material**, including disease-duration-adjusted logistic models, models including both thrombocytopenia strata, an extended Cox model adjusted for baseline thrombotic history and treatment variables, descriptive classification of incident and recurrent events, and exploratory Cox models for recurrent any thrombosis. Missing covariate data were handled using complete-case analysis, and no imputation was performed. All tests were two-sided, and *P* < 0.05 was considered statistically significant. Analyses were performed using SPSS version 26.0 and R version 4.3.1.

## Results

### Baseline characteristics according to platelet strata

A total of 313 patients with APS were included in the two-center baseline cohort: 51 in the PLT <50×10^9^/L group, 57 in the PLT 50–<100×10^9^/L group, and 205 in the PLT ≥100×10^9^/L group. Baseline demographic characteristics, cardiovascular risk factors, and clinical manifestations are summarized in **Table 1**.

**Table 1:** Baseline characteristics according to platelet strata in the combined APS cohort.

Characteristics	Overall (N = 313)	PLT <50×10^9^/L (n = 51)	PLT 50–<100×10^9^/L (n = 57)	PLT ≥100×10^9^/L (n = 205)	*P*-value
General information					
Study center, n (%)	RJ 254 (81.2) NT 59 (18.8)	RJ 47 (92.2) NT 4 (7.8)	RJ 49 (86.0) NT 8 (14.0)	RJ 158 (77.1) NT 47 (22.9)	0.028*
Age (years), median (25%–75%)	36.0 (29.0–54.0)	48.0 (36.0–59.5)^a^	40.0 (32.0–53.0)^ab^	34.0 (28.0–50.0)^b^	0.002*
Female, n (%)	241 (77.0)	40 (78.4)	42 (73.7)	159 (77.6)	0.799
Disease duration (months), median (25%–75%)	84.0 (24.0–144.0)	120.0 (78.0–187.5)^a^	72.0 (12.0–120.0)^b^	72.0 (12.0–144.0)^b^	<0.001*
BMI, kg/m^2^, median (25%–75%)	23.4 (20.7–25.7)	23.8 (21.6–26.0)	23.9 (21.5–26.6)	23.0 (20.3–25.0)	0.056
Primary APS, n (%)	129 (41.2)	21 (41.2)	25 (43.9)	83 (40.5)	0.901
SLE-associated APS, n (%)	171 (54.6)	30 (58.8)	30 (52.6)	111 (54.1)	0.789
Other APS subtype, n (%)	13 (4.2)	0 (0.0)	2 (3.5)	11 (5.4)	0.220
Hypertension, n (%)	71 (22.7)	19 (37.3)^a^	14 (24.6)^ab^	38 (18.5)^b^	0.016*
Diabetes mellitus, n (%)	22 (7.0)	10 (19.6)^a^	0 (0.0)^b^	12 (5.9)^b^	<0.001*
Hyperlipidemia, n (%)	70 (22.4)	14 (27.5)	13 (22.8)	43 (21.0)	0.608
Smoking, n (%)	8 (2.6)	2 (3.9)	1 (1.8)	5 (2.4)	0.763
aGAPSS score, median (25%–75%)	9 (5–13)	13 (9–14)^a^	9 (5–13)^ab^	9 (4–13)^b^	0.002*
GAPSS score, median (25%–75%)	12 (7–16)	13 (11–16)^a^	12 (8–16)^ab^	11 (7–16)^b^	0.004*
Clinical features at baseline					
Bleeding event, n (%)	8 (2.6)	3 (5.9)	3 (5.3)	2 (1.0)	0.050
Any thrombosis, n (%)	237 (75.7)	39 (76.5)	48 (84.2)	150 (73.2)	0.226
Arterial thrombosis, n (%)	132 (42.2)	34 (66.7)^a^	22 (38.6)^b^	76 (37.1)^b^	<0.001*
Cerebral infarction, n (%)	115 (36.7)	32 (62.7)^a^	19 (33.3)^b^	64 (31.2)^b^	<0.001*
Myocardial infarction, n (%)	7 (2.2)	1 (2.0)	3 (5.3)	3 (1.5)	0.227
Visceral arterial thrombosis, n (%)	8 (2.6)	4 (7.8)	1 (1.8)	3 (1.5)	0.032*
Venous thrombosis, n (%)	151 (48.2)	19 (37.3)^a^	39 (68.4)^b^	93 (45.4)^a^	0.002*
Deep venous thrombosis, n (%)	97 (31.0)	13 (25.5)	22 (38.6)	62 (30.2)	0.314
Pulmonary embolism, n (%)	56 (17.9)	3 (5.9)^a^	15 (26.3)^b^	38 (18.5)^ab^	0.020*
Visceral venous thrombosis, n (%)	19 (6.1)	5 (9.8)^ab^	7 (12.3)^a^	7 (3.4)^b^	0.022*
Cranial venous sinus thrombosis, n (%)	13 (4.2)	0 (0.0)^a^	8 (14.0)^b^	5 (2.4)^a^	<0.001*
Obstetric morbidity, n (%)**†**	53 (22.0)	8 (20.0)	7 (16.7)	38 (23.9)	0.570
Isolated ITP, n (%)	13 (4.2)	8 (15.7)^a^	5 (8.8)^a^	0 (0.0)^b^	<0.001*
Microangiopathy, n (%)	9 (2.9)	5 (9.8)^a^	1 (1.8)^ab^	3 (1.5)^b^	0.015*
Cardiac valve disease, n (%)	27 (8.6)	9 (17.6)	3 (5.3)	15 (7.3)	0.054
Autoimmune hemolytic anemia, n (%)	85 (27.2)	14 (27.5)^ab^	25 (43.9)^a^	46 (22.4)^b^	0.006*

**†**Obstetric morbidity was calculated among female patients. RJ, Renji Hospital; NT, Nantong First People’s Hospital. *P < 0.05 was considered statistically significant.

Compared with the PLT ≥100×10^9^/L group, patients with PLT <50×10^9^/L were older, had longer disease duration, and had higher frequencies of hypertension and diabetes mellitus. The aGAPSS and GAPSS scores were also higher in this group. Sex, body mass index, APS subtype, hyperlipidemia, and smoking status did not differ significantly across platelet strata.

Baseline thrombotic phenotypes differed across platelet strata. Arterial thrombosis was most frequent in the PLT <50×10^9^/L group, with cerebral infarction comprising most arterial events; the respective frequencies were 66.7% and 62.7%. In contrast, venous thrombosis was most frequent in the PLT 50–<100×10^9^/L group, occurring in 68.4% of patients, with higher frequencies of pulmonary embolism, visceral venous thrombosis, and cranial venous sinus thrombosis. Isolated immune thrombocytopenia, microangiopathy, and autoimmune hemolytic anemia also differed significantly among the three platelet strata.

When assessed according to the 2023 ACR/EULAR APS classification criteria, the distributions of individual classification domains differed across platelet strata (**Supplementary Table 2**). Macrovascular arterial thrombosis was more frequent in the PLT <50×10^9^/L group than in the PLT 50–<100×10^9^/L and PLT ≥100×10^9^/L groups (overall *P* < 0.001). Microvascular manifestations were also more frequent in the PLT <50×10^9^/L group (overall *P* = 0.015), whereas macrovascular venous thromboembolism was most frequent in the PLT 50–<100×10^9^/L group (overall *P* = 0.002). The solid-phase aPL laboratory domain was more frequent in the PLT <50×10^9^/L group (overall *P* = 0.043). No statistically significant differences were observed in obstetric manifestations, cardiac valve manifestations, or the lupus anticoagulant domain.

### Laboratory data and antibody profiles

Laboratory and antibody profiles are summarized in **Table 2**. As expected, PLT differed significantly across the three groups. Compared with the PLT ≥100×10^9^/L group, both thrombocytopenia groups had lower hemoglobin and C3 levels, whereas C4 and CRP levels did not differ significantly. ESR was higher in the PLT 50–<100×10^9^/L group than in the reference group.

**Table 2:** Laboratory data and antibody profiles.

Characteristics	Overall (N = 313)	PLT <50×10^9^/L (n = 51)	PLT 50–<100×10^9^/L (n = 57)	PLT ≥100×10^9^/L (n = 205)	*P*-value
Laboratory features					
PLT, ×10^9^/L, median (25%–75%)	150.0 (71.0–232.0)	30.0 (18.5–39.5)^a^	71.0 (62.0–84.0)^b^	207.0 (151.0–254.0)^c^	<0.001*
Hemoglobin, g/L, median (25%–75%)	113.0 (95.0–130.0)	99.0 (83.5–121.0)^a^	101.0 (92.0–119.0)^a^	118.0 (102.0–132.0)^b^	<0.001*
C3, g/L, median (25%–75%)	0.88 (0.66–1.06)	0.81 (0.57–0.95)^a^	0.78 (0.62–1.00)^a^	0.92 (0.71–1.11)^b^	0.001*
C4, g/L, median (25%–75%)	0.16 (0.10–0.23)	0.15 (0.09–0.19)	0.15 (0.10–0.21)	0.16 (0.10–0.24)	0.201
ESR, mm/h, median (25%–75%)	25 (11–51)	23 (9–55)^ab^	38 (13–67)^a^	23 (12–44)^b^	0.021*
CRP, mg/L, median (25%–75%)	2.53 (0.50–9.20)	3.85 (0.50–8.41)	4.20 (0.50–22.01)	1.92 (0.50–8.20)	0.062
Antibody profiles					
LA positivity, n (%)	263 (84.0)	46 (90.2)	50 (87.7)	167 (81.5)	0.220
Any aCL positivity, n (%)	170 (54.3)	37 (72.5)^a^	31 (54.4)^ab^	102 (49.8)^b^	0.014*
aCL IgA positivity, n (%)	78 (24.9)	9 (17.6)	16 (28.1)	53 (25.9)	0.399
aCL IgM positivity, n (%)	64 (20.4)	15 (29.4)	9 (15.8)	40 (19.5)	0.184
aCL IgG positivity, n (%)	126 (40.3)	28 (54.9)^a^	25 (43.9)^ab^	73 (35.6)^b^	0.035*
Any anti-β2GPI positivity, n (%)	184 (58.8)	33 (64.7)	34 (59.6)	117 (57.1)	0.605
anti-β2GPI IgM positivity, n (%)	150 (47.9)	29 (56.9)	26 (45.6)	95 (46.3)	0.375
anti-β2GPI IgG positivity, n (%)	133 (42.5)	22 (43.1)	30 (52.6)	81 (39.5)	0.207
Double-positive aPL, n (%)	83 (26.5)	15 (29.4)	15 (26.3)	53 (25.9)	0.875
Triple-positive aPL, n (%)	120 (38.3)	26 (51.0)	24 (42.1)	70 (34.1)	0.070
Any anti-PS/PT positivity, n (%)	205 (65.5)	34 (66.7)	40 (70.2)	131 (63.9)	0.666
anti-PS/PT IgM positivity, n (%)	171 (54.6)	27 (52.9)	31 (54.4)	113 (55.1)	0.961
anti-PS/PT IgG positivity, n (%)	144 (46.0)	30 (58.8)^a^	33 (57.9)^a^	81 (39.5)^b^	0.006*
Any anti-platelet antibody positivity, n (%)**†**	72 (61.5)	24 (68.6)	20 (71.4)	28 (51.9)	0.133
anti-GP IX antibody positivity, n (%)**†**	23 (19.7)	12 (34.3)^a^	6 (21.4)^ab^	5 (9.3)^b^	0.014*
anti-GP IIb antibody positivity, n (%)**†**	58 (49.6)	19 (54.3)^ab^	19 (67.9)^a^	20 (37.0)^b^	0.024*
ANA positivity, n (%)	236 (75.4)	38 (74.5)	41 (71.9)	157 (76.6)	0.761
anti-dsDNA positivity, n (%)	117 (37.4)	14 (27.5)	19 (33.3)	84 (41.0)	0.159

†Anti-platelet antibody percentages were calculated among patients with available tests (overall n=117; PLT <50×10^9^/L, n=35; PLT 50–<100×10^9^/L, n=28; PLT ≥100×10^9^/L, n=54). *P < 0.05 was considered statistically significant.

Regarding antibody profiles, the PLT <50×10^9^/L group had higher frequencies of any aCL positivity and aCL IgG positivity than the reference group. Anti-PS/PT IgG positivity was more frequent in both thrombocytopenia groups than in the PLT ≥100×10^9^/L group. No significant differences were observed in LA positivity, anti-β2GPI positivity, double-positive aPL, or triple-positive aPL, although triple-positive aPL tended to be more frequent in the PLT <50×10^9^/L group. Among patients with available anti-platelet antibody testing, anti-GP IX and anti-GP IIb antibody positivity differed significantly across platelet strata.

### Baseline treatment characteristics

Baseline treatment characteristics are summarized in **Table 3**. Low-molecular-weight heparin was more frequently used in both thrombocytopenia groups than in the PLT ≥100×10^9^/L group. Glucocorticoid use was highest in the PLT <50×10^9^/L group, and anti-CD20 therapy was more common in this group than in the reference group. No significant differences were observed in antiplatelet therapy, overall anticoagulation, warfarin, direct oral anticoagulants, hydroxychloroquine, or conventional immunosuppressants.

**Table 3:** Baseline treatment characteristics.

Characteristics	Overall (N = 313)	PLT <50×10^9^/L (n = 51)	PLT 50–<100×10^9^/L (n = 57)	PLT ≥100×10^9^/L (n = 205)	*P*-value
Antiplatelet therapy, n (%)	75 (24.0)	12 (23.5)	7 (12.3)	56 (27.3)	0.063
Anticoagulation, n (%)	245 (78.3)	37 (72.5)	51 (89.5)	157 (76.6)	0.063
Warfarin, n (%)	150 (47.9)	20 (39.2)	32 (56.1)	98 (47.8)	0.213
Low-molecular-weight heparin, n (%)	157 (50.2)	32 (62.7)^a^	36 (63.2)^a^	89 (43.4)^b^	0.004*
DOACs, n (%)	53 (16.9)	3 (5.9)	10 (17.5)	40 (19.5)	0.067
Glucocorticoids, n (%)	267 (85.3)	50 (98.0)^a^	51 (89.5)^ab^	166 (81.0)^b^	0.005*
Hydroxychloroquine, n (%)	207 (66.1)	28 (54.9)	39 (68.4)	140 (68.3)	0.180
Conventional immunosuppressants, n (%)	104 (33.2)	14 (27.5)	21 (36.8)	69 (33.7)	0.571
Anti-CD20 therapy, n (%)	110 (35.1)	25 (49.0)^a^	24 (42.1)^ab^	61 (29.8)^b^	0.017*
Belimumab, n (%)	9 (2.9)	1 (2.0)	1 (1.8)	7 (3.4)	0.732
Telitacicept, n (%)	14 (4.5)	5 (9.8)	0 (0.0)	9 (4.4)	0.048*

DOACs included rivaroxaban and edoxaban. Conventional immunosuppressants included mycophenolate mofetil, methotrexate, cyclophosphamide, tacrolimus, cyclosporine, azathioprine, sirolimus, and thalidomide. Anti-CD20 therapy included rituximab, obinutuzumab, and ofatumumab. *P < 0.05 was considered statistically significant.

### Association between severe thrombocytopenia and baseline thrombotic phenotypes

Multivariable logistic regression analyses were performed in 300 patients with primary APS or SLE-associated APS and complete covariate data, after excluding 13 patients with other APS subtypes (**Table 4**). After adjustment for age, sex, center, diabetes mellitus, smoking, APS subtype, and aGAPSS, PLT <50×10^9^/L remained associated with a higher prevalence of arterial thrombosis (aOR 2.16, 95% CI 1.07–4.35, P = 0.032) and cerebral infarction (aOR 2.37, 95% CI 1.18–4.73, P = 0.015), but not with any thrombosis or venous thrombosis. These associations remained consistent after additional adjustment for disease duration (arterial thrombosis: aOR 2.28, 95% CI 1.12–4.64, P = 0.023; cerebral infarction: aOR 2.47, 95% CI 1.22–4.97, P = 0.011). In contrast, PLT 50–<100×10^9^/L was associated with a higher prevalence of venous thrombosis in the disease-duration-adjusted model (aOR 2.99, 95% CI 1.49–6.01, *P* = 0.002), but not with arterial thrombosis or cerebral infarction (**Supplementary Table 3**).

**Table 4:** Baseline multivariable logistic regression analyses for prevalent thrombotic phenotypes.

Outcome	Events/N	Multivariable analysis, aOR (95% CI), *P*-value		
		Model 1	Model 2	Model 3
Any thrombosis	224/300	0.76 (0.34–1.68), *P* = 0.495	0.70 (0.31–1.57), *P* = 0.388	0.80 (0.35–1.84), *P* = 0.602
Arterial thrombosis	130/300	2.42 (1.22–4.80), *P* = 0.011*	2.31 (1.15–4.62), *P* = 0.018*	2.16 (1.07–4.35), *P* = 0.032*
Cerebral infarction	114/300	2.61 (1.33–5.12), *P* = 0.005*	2.44 (1.23–4.85), *P* = 0.011*	2.37 (1.18–4.73), *P* = 0.015*
Venous thrombosis	138/300	0.65 (0.33–1.27), *P* = 0.205	0.69 (0.35–1.35), *P* = 0.276	0.79 (0.39–1.59), *P* = 0.509

Estimates represent PLT <50×10^9^/L versus PLT ≥100×10^9^/L. PLT ≥100×10^9^/L was used as the reference. *P < 0.05 was considered statistically significant.

In extended multivariable models simultaneously including platelet strata, cardiac valve disease, and microvascular disease, PLT <50×10^9^/L remained associated with arterial thrombosis (aOR 2.16, 95% CI 1.04–4.48, *P* = 0.039) and cerebral infarction (aOR 2.37, 95% CI 1.16–4.84, *P* = 0.017). Cardiac valve disease was also associated with arterial thrombosis (aOR 2.80, 95% CI 1.11–7.09, *P* = 0.029), whereas microvascular disease was not statistically significant (**Supplementary Table 4**). After excluding patients with cardiac valve disease or microvascular disease (n=265), the associations of PLT <50×10^9^/L with arterial thrombosis (aOR 1.51, 95% CI 0.69–3.31, *P* = 0.303) and cerebral infarction (aOR 1.68, 95% CI 0.77–3.67, *P* = 0.196) were attenuated and no longer statistically significant. In the restricted cohort, PLT 50–<100×10^9^/L remained associated with venous thrombosis (aOR 2.36, 95% CI 1.16–4.81, *P* = 0.018; **Supplementary Table 5**).

Triple-positive aPL profile was present in 26 of 51 patients (51.0%) with PLT <50×10^9^/L, 24 of 57 patients (42.1%) with PLT 50–<100×10^9^/L, and 70 of 205 patients (34.1%) with PLT ≥100×10^9^/L (overall *P* = 0.070; **Table 2**). In alternative multivariable models replacing aGAPSS with triple-positive aPL profile and separately adjusting for hypertension and hyperlipidemia, PLT <50×10^9^/L remained associated with arterial thrombosis (aOR 2.31, 95% CI 1.13–4.71, *P* = 0.022) and cerebral infarction (aOR 2.47, 95% CI 1.23–4.97, *P* = 0.011), whereas PLT 50–<100×10^9^/L remained associated with venous thrombosis (aOR 2.78, 95% CI 1.40–5.52, *P* = 0.003; **Supplementary Table 6**). In Cox sensitivity models additionally adjusted for triple-positive aPL profile, PLT <50×10^9^/L remained associated with any thrombotic event (HR 3.32, 95% CI 1.56–7.08, *P* = 0.002), arterial thrombotic events (HR 3.97, 95% CI 1.40–11.23, *P* = 0.009), and cerebral infarction (HR 4.10, 95% CI 1.44–11.66, *P* = 0.008), but not venous-related thrombotic events (HR 1.19, 95% CI 0.40–3.57, *P* = 0.755; **Supplementary Table 7**).

### Follow-up thrombotic events

**Figure 2:**
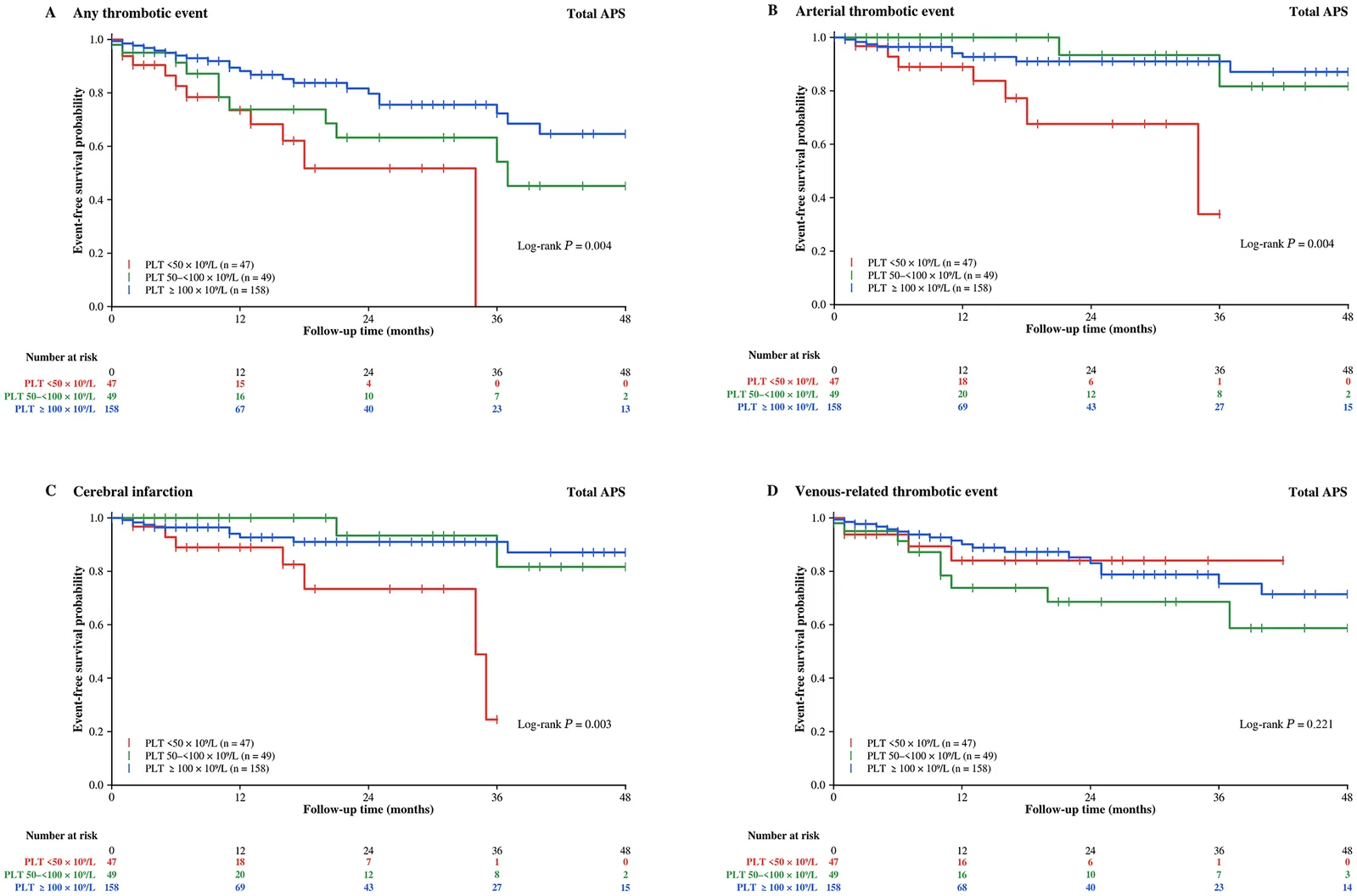
Kaplan–Meier curves for 48-month event-free survival according to platelet strata. **(A)** Any thrombotic event; **(B)** arterial thrombotic event; **(C)** cerebral infarction; **(D)** venous-related thrombotic event. Panels B and C overlap substantially because nearly all arterial thrombotic events were cerebral infarctions. One patient experienced a non-cerebral arterial thrombotic event at month 13 and subsequently developed cerebral infarction at month 35. P values were calculated using the log-rank test.

**Figure 3:**
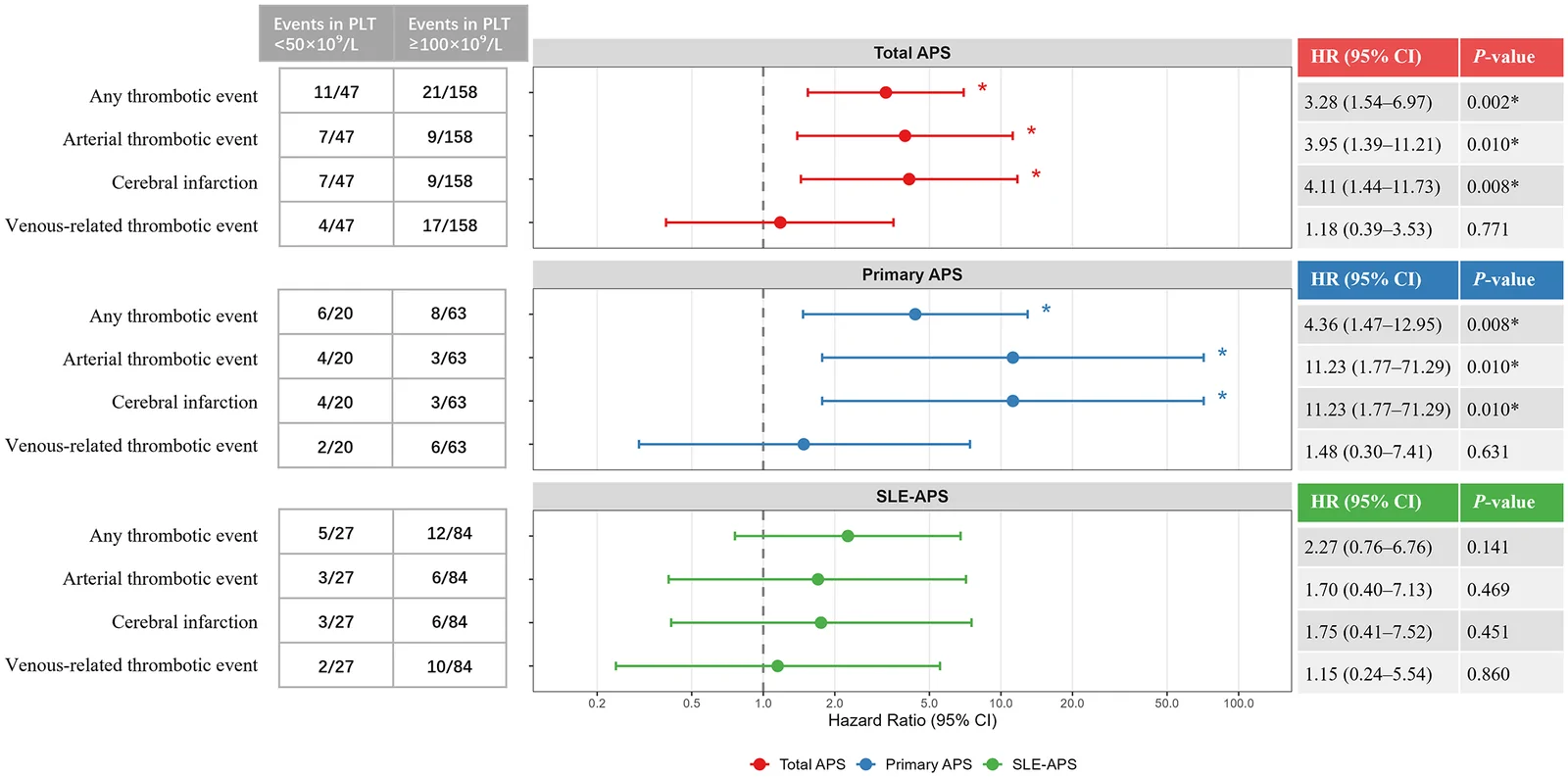
Multivariable Cox regression analyses of follow-up thrombotic events according to severe thrombocytopenia. Estimates represent PLT <50×10^9^/L versus PLT ≥100×10^9^/L. PLT ≥100×10^9^/L was used as the reference group. *P < 0.05 was considered statistically significant.

Follow-up analyses were conducted in the Renji cohort, with follow-up truncated at 48 months. Kaplan–Meier analyses showed significant differences across platelet strata for any thrombotic event (log-rank *P* = 0.004), arterial thrombotic event (log-rank *P* = 0.004), and cerebral infarction (log-rank *P* = 0.003), but not for venous-related thrombotic event (log-rank *P* = 0.221; **Figure 2**). The arterial thrombotic event and cerebral infarction curves overlapped substantially because most arterial-event times coincided with cerebral infarction events. In the total APS cohort, one patient experienced a non-cerebral arterial thrombotic event at month 13 and subsequently developed cerebral infarction at month 35 (**Figures 2B, C**). In the primary APS subgroup, all eight arterial thrombotic events were cerebral infarctions involving the same patients and identical event times (**Supplementary Figures 1B, C**). In the total APS cohort, PLT <50×10^9^/L was associated with higher risks of any thrombotic event (HR 3.28, 95% CI 1.54–6.97, *P* = 0.002), arterial thrombotic event (HR 3.95, 95% CI 1.39–11.21, *P* = 0.010), and cerebral infarction (HR 4.11, 95% CI 1.44–11.73, *P* = 0.008), but not venous-related thrombotic events (**Figure 3**). These findings remained directionally consistent in the extended Cox sensitivity model adjusted for baseline thrombotic history and treatment variables: any thrombotic event (HR 2.73, 95% CI 1.21–6.15, *P* = 0.016), arterial thrombotic event (HR 3.90, 95% CI 1.19–12.82, *P* = 0.025), cerebral infarction (HR 4.15, 95% CI 1.26–13.65, *P* = 0.019), and venous-related thrombotic event (HR 1.08, 95% CI 0.34–3.44, *P* = 0.901; **Supplementary Table 8**).

Severe outcomes were uncommon during the 48-month follow-up. Among the 254 patients in the Renji follow-up cohort, four cases of CAPS (1.6%) and one death (0.4%) were recorded. CAPS occurred in one patient with PLT <50×10^9^/L, one patient with PLT 50–<100×10^9^/L, and two patients with PLT ≥100×10^9^/L. The single death occurred in the PLT 50–<100×10^9^/L group. Because of the very small number of events, mortality and CAPS were reported descriptively, without formal between-group comparisons or multivariable analyses.

Follow-up events were additionally separated into incident and recurrent events according to the corresponding baseline thrombotic history (**Supplementary Table 9**). Among 213 patients with any thrombosis at baseline, 32 recurrent any thrombotic events occurred during follow-up: 9 of 38 patients (23.7%) with PLT <50×10^9^/L, 10 of 44 patients (22.7%) with PLT 50–<100×10^9^/L, and 13 of 131 patients (9.9%) with PLT ≥100×10^9^/L. In the exploratory Cox model adjusted for age, sex, and APS subtype, PLT <50×10^9^/L was associated with a higher risk of recurrent thrombosis (HR 5.15, 95% CI 2.11–12.60, P<0.001), and PLT 50–<100×10^9^/L was also associated with increased recurrence risk (HR 3.09, 95% CI 1.34–7.14, *P* = 0.008), compared with PLT ≥100×10^9^/L (**Supplementary Table 10**). Because only 32 recurrent events were observed, these analyses were considered exploratory and hypothesis-generating.

Among 140 patients without arterial thrombosis at baseline, nine first-ever arterial events occurred: 1/14 (7.1%) in the PLT <50×10^9^/L group, 1/30 (3.3%) in the PLT 50–<100×10^9^/L group, and 7/96 (7.3%) in the PLT ≥100×10^9^/L group. Event-free survival did not differ across platelet strata (log-rank *P* = 0.529). Given the limited number of events, adjusted Cox modeling was not performed (**Supplementary Table 11**).

Exploratory subgroup analyses showed directionally stronger point estimates in primary APS. PLT <50×10^9^/L was associated with any thrombotic event and arterial thrombotic events, particularly cerebral infarction, in primary APS, whereas no statistically significant association was observed in SLE-associated APS. In reduced extended subgroup sensitivity models, the estimates in primary APS were HR 4.83 (95% CI 1.37–17.12, *P* = 0.015) for any thrombotic event and HR 17.05 (95% CI 1.50–194.38, *P* = 0.022) for arterial thrombotic events; the estimate for cerebral infarction was identical. However, the extremely wide confidence intervals reflected the limited number of events and substantial statistical uncertainty. Corresponding estimates remained non-significant in SLE-associated APS after additional adjustment (**Supplementary Table 12**). These subgroup analyses and the corresponding Kaplan–Meier curves in **Supplementary Figures 1** and **2** were considered exploratory and should not be interpreted as establishing definitive differences between APS subtypes.

## Discussion

In this two-center APS cohort, severe thrombocytopenia, defined as PLT <50×10^9^/L, was associated with a higher prevalence of arterial thrombotic manifestations at baseline and a greater risk of subsequent arterial thrombotic events, particularly cerebral infarction. Patients with PLT <50×10^9^/L were older and had longer disease duration, higher aGAPSS and GAPSS scores, and a greater burden of cardiovascular comorbidities than patients with PLT ≥100×10^9^/L. Although the associations persisted after multivariable adjustment and additional sensitivity analyses, residual confounding cannot be excluded. Severe thrombocytopenia may therefore represent a clinical marker of cumulative disease burden, previous vascular injury, or overall APS severity rather than a biologically independent thrombotic phenotype. In addition, because the baseline logistic analyses were cross-sectional, the temporal relationship between thrombocytopenia and prevalent thrombotic manifestations could not be established. Severe thrombocytopenia may have preceded thrombosis, developed as part of cumulative APS activity, or occurred after previous thrombotic events and subsequent treatment. Accordingly, the baseline findings demonstrate associations rather than causality.

Across the APS cohort, the relative distribution of individual weighted clinical and laboratory domains differed across platelet strata. The enrichment of arterial, microvascular, and solid-phase aPL domains among patients with severe thrombocytopenia further illustrates clinical heterogeneity within a uniformly classified APS population. However, the hematological-domain comparison is partly inherent to the platelet-based exposure definition and should be interpreted cautiously.

Thrombocytopenia has traditionally been regarded as an immune thrombocytopenia-like manifestation or a bleeding-related concern in APS. However, accumulating evidence indicates that APS-related thrombocytopenia does not necessarily imply a low thrombotic risk (6–8). Previous studies have reported associations between thrombocytopenia and thrombotic events, pregnancy morbidity, and non-criteria manifestations in APS (23). In a prospective primary APS cohort, thrombocytopenia defined as PLT <100×10^9^/L was independently associated with thrombotic events and severe extra-criteria manifestations (7). Importantly, PLT <50×10^9^/L should not be interpreted as an established APS-specific arterial-risk cutoff. Our incremental contribution is the severity-based stratification of platelet count beyond the conventional binary PLT <100×10^9^/L definition, with the strongest association concentrated in the PLT <50×10^9^/L group and predominantly involving arterial thrombotic manifestations, particularly cerebral infarction.

A notable finding of this study was the divergent distribution of arterial and venous thrombotic manifestations across platelet strata. Patients with PLT <50×10^9^/L showed enrichment of arterial thrombosis, particularly cerebral infarction, whereas venous thrombosis was most frequent in the PLT 50–<100×10^9^/L group. This pattern was supported by sensitivity logistic models including both thrombocytopenia strata and additional adjustment for disease duration: severe thrombocytopenia remained associated with a higher prevalence of arterial thrombosis, particularly cerebral infarction, while moderate thrombocytopenia was associated with a higher prevalence of venous thrombosis. These findings suggest that different degrees of thrombocytopenia may be associated with different distributions of arterial and venous thrombotic manifestations. However, they should not be interpreted as evidence of biologically distinct phenotypes, because differences in disease duration, comorbidities, antibody profiles, previous vascular injury, and treatment exposure may have contributed to the observed associations. One possible explanation is that arterial thrombosis, particularly cerebral infarction, may be more closely related to platelet activation, endothelial injury, complement activation, and local thromboinflammation, whereas venous thrombosis may be more influenced by coagulation activation and systemic hypercoagulability (24–26). The present observational study was not designed to establish these mechanisms.

The antibody and laboratory findings provide biological plausibility for an association between thrombocytopenia and immune-thrombotic activation. Patients with severe thrombocytopenia had higher frequencies of any aCL positivity and aCL IgG positivity, while anti-PS/PT IgG positivity was more frequent in both thrombocytopenia groups than in the reference group. This is consistent with previous evidence linking anti-PS/PT antibodies to thrombotic manifestations in APS (17, 27, 28). In addition, lower hemoglobin and C3 levels in the thrombocytopenia groups, together with differences in anti-GP IX and anti-GP IIb antibody positivity among patients with available testing, suggest that thrombocytopenia in APS may reflect broader immune activation, complement consumption, and platelet-directed autoimmunity rather than isolated peripheral platelet destruction. Importantly, APS-associated thrombocytopenia is mechanistically heterogeneous and may reflect immune-mediated platelet destruction, microangiopathic platelet consumption, or coexistence with other autoimmune cytopenias; PLT <50×10^9^/L should therefore not be considered a biologically uniform entity. Mechanistically, aPLs may interact with platelets and endothelial cells, promote platelet activation and consumption, and amplify thromboinflammatory responses through complement activation and neutrophil extracellular trap formation (14). Nevertheless, the present data cannot determine whether thrombocytopenia directly contributes to thrombosis or instead reflects broader immune-thrombotic activity and cumulative disease severity.

Cardiac valve disease and microvascular manifestations may represent components of the same high-risk immune-thrombotic profile as severe thrombocytopenia. Although PLT <50×10^9^/L remained associated with arterial thrombosis, particularly cerebral infarction, in models simultaneously adjusting for these manifestations, the associations were attenuated after patients with cardiac valve or microvascular disease were excluded. This attenuation suggests that these manifestations may partly contribute to the observed association with arterial thrombosis, although the reduced sample size and event counts in the restricted cohort should also be considered. Similarly, adjustment for triple-positive aPL status did not materially attenuate the platelet-stratum estimates. These complementary analyses support interpreting severe thrombocytopenia as one component of a broader high-risk APS profile rather than as an isolated causal determinant.

The association between severe thrombocytopenia and follow-up thrombotic events appeared more evident in primary APS than in SLE-associated APS. However, these subgroup analyses were exploratory and were not adequately powered to establish differential effects by APS subtype. The very wide confidence intervals, including an HR of 17.05 with a 95% CI of 1.50–194.38, indicate substantial statistical uncertainty. The lack of statistical significance in SLE-associated APS should not be interpreted as evidence of no risk. In SLE-associated APS, thrombocytopenia may be influenced by lupus activity, immune complex-mediated complement activation, nephritis, cytopenias, and immunosuppressive treatment, which may reduce its specificity as a marker of APS-related arterial thrombotic risk (29–31). The apparent differences between primary APS and SLE-associated APS therefore require confirmation in larger cohorts.

Exploratory analyses restricted to patients with baseline thrombosis suggested that both severe and moderate thrombocytopenia were associated with an increased risk of recurrent thrombosis. This observation suggests that platelet-count severity may carry prognostic information not only for overall follow-up thrombosis but also for recurrence. Nevertheless, the recurrence analysis was based on only 32 events, resulting in limited precision and a risk of model overfitting. Residual confounding by baseline thrombotic burden, treatment indication, disease activity, and changes in platelet counts during follow-up cannot be excluded. These findings should therefore be considered hypothesis-generating and require validation in larger prospective cohorts specifically designed to evaluate recurrent thrombosis.

Recent studies further support the prognostic relevance of thrombocytopenia in APS. In a 2025 single-center cohort of 250 patients, thrombocytopenia was associated with recurrent thrombosis (HR 2.57, 95% CI 1.01–6.02) (32). A prospective cluster analysis identified clinically distinct subgroups of primary APS-associated thrombocytopenia with different five-year outcomes, including thrombosis, microvascular disease, and valvular manifestations (33). A 2026 retrospective study of 432 patients similarly found thrombocytopenia to be associated with a broader burden of thrombotic, valvular, and CAPS manifestations (34). These observations are consistent with our interpretation that severe thrombocytopenia may mark a broader high-risk APS profile rather than acting as an isolated causal factor.

This study has several clinical implications. First, PLT <50×10^9^/L should not be interpreted solely as a bleeding-related marker in APS, because patients with severe thrombocytopenia may still have a substantial burden of arterial thrombotic manifestations, particularly cerebral infarction. Second, platelet-count stratification may provide additional clinical risk information beyond the conventional binary definition of thrombocytopenia as PLT <100×10^9^/L. Third, arterial and venous thrombotic risks should be assessed separately, as severe and moderate thrombocytopenia were associated with different distributions of thrombotic manifestations. These findings may help refine clinical risk assessment in APS, although antithrombotic treatment decisions should continue to balance thrombotic and bleeding risks on an individual basis (35–37).

Several limitations should be acknowledged. First, this was an observational study, and residual confounding cannot be fully excluded. Patients with severe thrombocytopenia were older and had longer disease duration, higher aGAPSS scores, and more cardiovascular comorbidities; severe thrombocytopenia may therefore have functioned as a surrogate marker of cumulative disease burden, previous vascular injury, or overall APS severity. Second, the baseline logistic analyses were cross-sectional and could not establish whether thrombocytopenia preceded or followed the prevalent thrombotic manifestations. Only nine first-ever arterial events occurred among patients without baseline arterial thrombosis, limiting evaluation of incident arterial risk. Third, baseline PLT was obtained before treatment initiation, escalation, or modification during the index hospitalization; however, some patients had already been receiving maintenance glucocorticoid, immunosuppressive, biologic, or antithrombotic treatment before admission. Prior treatment exposure may therefore have influenced the measured baseline platelet count. Platelet strata were also defined using a single baseline measurement, and dynamic changes in platelet counts, duration of thrombocytopenia, and treatment response were not modeled as time-varying exposures. Fourth, treatment patterns differed across platelet strata and may have been influenced by thrombocytopenia severity, baseline thrombotic history, and disease activity. Although baseline treatment variables were included in sensitivity analyses, residual confounding by indication could not be eliminated. Fifth, standardized longitudinal follow-up data were available only for the Renji cohort; baseline differences between centers, including disease duration, thrombotic burden, platelet-stratum distribution, and treatment exposures, may limit the generalizability of the longitudinal findings (**Supplementary Table 13**). Finally, the number of follow-up arterial and recurrent events was limited, especially in subgroup analyses, resulting in wide confidence intervals and a risk of overfitting. The subgroup and recurrence analyses should therefore be considered exploratory, and all findings require validation in larger prospective cohorts incorporating longitudinal platelet measurements and treatment changes. Mortality and CAPS were rare, with only one death and four CAPS cases recorded during follow-up. The study was therefore underpowered to assess whether platelet-count severity was associated with these general severity outcomes, and their distribution should be interpreted descriptively.

In conclusion, severe thrombocytopenia, defined as PLT <50×10^9^/L, was associated with a higher prevalence of arterial thrombotic manifestations at baseline and a greater risk of subsequent arterial thrombotic events, particularly cerebral infarction, in patients with APS. Platelet-count severity may provide additional information for clinical risk assessment. However, severe thrombocytopenia may also reflect cumulative disease burden and overall APS severity, and the observed associations should not be interpreted as evidence of an independent causal phenotype. Prospective studies incorporating longitudinal platelet measurements, disease activity, and cumulative treatment exposure are needed to confirm these findings.

## Data Availability

The individual-level datasets generated and analyzed during the current study are not publicly available because they contain potentially identifiable clinical information, and public disclosure was not covered by the ethics approval or participant consent. Deidentified data may be made available from the corresponding author upon reasonable request, subject to approval by the relevant ethics committees and institutional data-use requirements. The aggregate data supporting the conclusions of this article are provided in the article and **Supplementary Material**.
