# Supplementary material for: Severe thrombocytopenia is associated with arterial thrombotic manifestations in antiphospholipid syndrome: a two-center cohort study
Source: Front Immunol. 2026 Aug 26;17:1927065. doi: 10.3389/fimmu.2026.1927065 (PMC13553740; doi:10.3389/fimmu.2026.1927065)
Supplement: Supplementary file 1 [file Table1.docx]

**Supplementary Material**

This supplementary document provides additional analyses of platelet-count strata and thrombotic manifestations in antiphospholipid syndrome (APS). PLT ≥100×10⁹/L was used as the reference group in regression models.

The supplementary analyses include proportional-hazards testing; 2023 ACR/EULAR APS classification-domain analyses; baseline and Cox sensitivity analyses; incident and recurrent thrombotic-event analyses; a restricted analysis of first-ever arterial events; APS-subtype analyses; and a baseline center comparison. Analyses with limited event numbers were considered exploratory. All tests were two-sided, and *P*<0.05 was considered statistically significant.

**Supplementary Table S1. Proportional hazards assumption tests for the primary Cox models**

The proportional hazards assumption was assessed using Schoenfeld residuals in the primary Cox models for the total APS cohort.

| **Outcome** | **PLT <50×10⁹/L PH *P*-value** | **Global PH *P*-value** | **Interpretation** |
| --- | --- | --- | --- |
| Any thrombotic event | 0.897 | 0.247 | No major violation |
| Arterial thrombotic event | 0.141 | 0.357 | No major violation |
| Cerebral infarction | 0.083 | 0.243 | No major violation |
| Venous-related thrombotic event | 0.142 | 0.347 | No major violation |

Data are shown as *P* values from Schoenfeld residual tests. The primary Cox models for the total APS cohort were adjusted for age, sex, and APS subtype. PLT ≥100×10⁹/L was used as the reference group. *P* value >0.05 indicates no evidence of a major violation of the proportional hazards assumption. PH, proportional hazards.

**Supplementary Table S2. Distribution of the 2023 ACR/EULAR APS classification domains according to platelet strata**

| **2023 ACR/EULAR domain** | **Overall** | **PLT <50×10⁹/L** | **PLT 50–<100×10⁹/L** | **PLT ≥100×10⁹/L** | ***P*-value** |
| --- | --- | --- | --- | --- | --- |
| Macrovascular venous thromboembolism | 151/313 (48.2%) | 19/51 (37.3%) | 39/57 (68.4%) | 93/205 (45.4%) | 0.002* |
| Macrovascular arterial thrombosis | 132/313 (42.2%) | 34/51 (66.7%) | 22/57 (38.6%) | 76/205 (37.1%) | <0.001* |
| Microvascular manifestations | 9/313 (2.9%) | 5/51 (9.8%) | 1/57 (1.8%) | 3/205 (1.5%) | 0.015* |
| Obstetric manifestations | 53/241 (22.0%) | 8/40 (20.0%) | 7/42 (16.7%) | 38/159 (23.9%) | 0.570 |
| Cardiac valve manifestations | 27/313 (8.6%) | 9/51 (17.6%) | 3/57 (5.3%) | 15/205 (7.3%) | 0.054 |
| Hematological domain | 130/313 (41.5%) | 37/51 (72.5%) | 57/57 (100.0%) | 36/205 (17.6%) | <0.001* |
| Lupus anticoagulant laboratory domain | 263/313 (84.0%) | 46/51 (90.2%) | 50/57 (87.7%) | 167/205 (81.5%) | 0.220 |
| Solid-phase aPL laboratory domain | 218/313 (69.6%) | 43/51 (84.3%) | 39/57 (68.4%) | 136/205 (66.3%) | 0.043* |

Data are presented as n/N (%). Overall *P* values were calculated using Pearson’s *χ²* test or the Fisher–Freeman–Halton exact test, as appropriate. Holm-adjusted pairwise comparisons showed that macrovascular venous thromboembolism was more frequent in the PLT 50–<100×10⁹/L group than in both other groups (both adjusted *P*=0.006); macrovascular arterial thrombosis was more frequent in the PLT <50×10⁹/L group than in the PLT 50–<100×10⁹/L group (adjusted *P*=0.008) and the PLT ≥100×10⁹/L group (adjusted *P*<0.001); microvascular manifestations were more frequent in the PLT <50×10⁹/L group than in the PLT ≥100×10⁹/L group (adjusted *P*=0.027); and the solid-phase aPL domain was more frequent in the PLT <50×10⁹/L group than in the PLT ≥100×10⁹/L group (adjusted *P*=0.048).

aCL, anticardiolipin antibody; anti-β2GPI, anti-β2-glycoprotein I antibody; aPL, antiphospholipid antibody; APS, antiphospholipid syndrome; PLT, platelet count. **P*<0.05 was considered statistically significant.

**Supplementary Table S3. Baseline multivariable logistic regression analyses including both thrombocytopenia strata and disease-duration adjustment**

| **Outcome** | **Events/N** | **Model 3** | **Model 3** | **Model 3 + disease duration** | **Model 3 + disease duration** |
| --- | --- | --- | --- | --- | --- |
|  |  | **PLT <50×10⁹/L** | **PLT 50–<100×10⁹/L** | **PLT <50×10⁹/L** | **PLT 50–<100×10⁹/L** |
| Any thrombosis | 224/300 | 0.80 (0.35–1.84), *P*=0.602 | 1.76 (0.74–4.20), *P*=0.201 | 0.77 (0.34–1.78), *P*=0.544 | 1.82 (0.76–4.36), *P*=0.179 |
| Arterial thrombosis | 130/300 | 2.16 (1.07–4.35), *P*=0.032* | 0.86 (0.45–1.66), *P*=0.655 | 2.28 (1.12–4.64), *P*=0.023* | 0.83 (0.43–1.61), *P*=0.575 |
| Cerebral infarction | 114/300 | 2.37 (1.18–4.73), *P*=0.015* | 0.88 (0.45–1.74), *P*=0.720 | 2.47 (1.22–4.97), *P*=0.011* | 0.85 (0.43–1.69), *P*=0.646 |
| Venous thrombosis | 138/300 | 0.79 (0.39–1.59), *P*=0.509 | 2.79 (1.40–5.55), *P*=0.004* | 0.72 (0.35–1.47), *P*=0.370 | 2.99 (1.49–6.01), *P*=0.002* |

Data are shown as adjusted odds ratios (aORs) with 95% confidence intervals (CIs). Model 3 was adjusted for age, sex, center, diabetes mellitus, smoking, APS subtype, and aGAPSS. The disease-duration-adjusted model was further adjusted for disease duration. Both thrombocytopenia strata were included in the same model, and PLT ≥100×10⁹/L was used as the reference group. **P*<0.05 was considered statistically significant.

**Supplementary Table S4. Extended multivariable logistic regression models including platelet strata, cardiac valve disease, and microvascular disease**

| **Outcome** | **Predictor** | **Adjusted OR (95% CI)** | ***P*-value** |
| --- | --- | --- | --- |
| Any thrombosis | PLT <50 vs ≥100×10⁹/L | 0.79 (0.34–1.85) | 0.589 |
| Any thrombosis | PLT 50–<100 vs ≥100×10⁹/L | 1.80 (0.75–4.28) | 0.187 |
| Any thrombosis | Cardiac valve disease | 1.68 (0.57–4.95) | 0.350 |
| Any thrombosis | Microvascular disease | 0.86 (0.16–4.67) | 0.865 |
| Arterial thrombosis | PLT <50 vs ≥100×10⁹/L | 2.16 (1.04–4.48) | 0.039* |
| Arterial thrombosis | PLT 50–<100 vs ≥100×10⁹/L | 0.89 (0.46–1.73) | 0.737 |
| Arterial thrombosis | Cardiac valve disease | 2.80 (1.11–7.09) | 0.029* |
| Arterial thrombosis | Microvascular disease | 0.70 (0.16–3.08) | 0.641 |
| Cerebral infarction | PLT <50 vs ≥100×10⁹/L | 2.37 (1.16–4.84) | 0.017* |
| Cerebral infarction | PLT 50–<100 vs ≥100×10⁹/L | 0.89 (0.45–1.74) | 0.729 |
| Cerebral infarction | Cardiac valve disease | 1.16 (0.48–2.81) | 0.737 |
| Cerebral infarction | Microvascular disease | 0.88 (0.20–3.86) | 0.870 |
| Venous thrombosis | PLT <50 vs ≥100×10⁹/L | 0.77 (0.37–1.59) | 0.483 |
| Venous thrombosis | PLT 50–<100 vs ≥100×10⁹/L | 2.77 (1.39–5.52) | 0.004* |
| Venous thrombosis | Cardiac valve disease | 0.89 (0.37–2.17) | 0.802 |
| Venous thrombosis | Microvascular disease | 1.33 (0.31–5.67) | 0.700 |

Data are shown as adjusted odds ratios (aORs) with 95% confidence intervals. The analytic cohort comprised 300 patients with primary APS or SLE-associated APS. All models simultaneously included PLT <50×10⁹/L, PLT 50–<100×10⁹/L, cardiac valve disease, and microvascular disease and were adjusted for age, sex, study center, diabetes mellitus, smoking, APS subtype, and aGAPSS. PLT ≥100×10⁹/L was the reference group. **P*<0.05 was considered statistically significant.

**Supplementary Table S5. Sensitivity analysis excluding patients with cardiac valve disease or microvascular disease while retaining the original three platelet strata**

| **Outcome** | **Events/N** | **Predictor** | **Adjusted OR (95% CI)** | ***P*-value** |
| --- | --- | --- | --- | --- |
| Any thrombosis | 196/265 | PLT <50 vs ≥100×10⁹/L | 0.45 (0.18–1.11) | 0.085 |
| Any thrombosis | 196/265 | PLT 50–<100 vs ≥100×10⁹/L | 1.73 (0.68–4.43) | 0.254 |
| Arterial thrombosis | 108/265 | PLT <50 vs ≥100×10⁹/L | 1.51 (0.69–3.31) | 0.303 |
| Arterial thrombosis | 108/265 | PLT 50–<100 vs ≥100×10⁹/L | 0.85 (0.43–1.69) | 0.636 |
| Cerebral infarction | 98/265 | PLT <50 vs ≥100×10⁹/L | 1.68 (0.77–3.67) | 0.196 |
| Cerebral infarction | 98/265 | PLT 50–<100 vs ≥100×10⁹/L | 0.86 (0.42–1.74) | 0.670 |
| Venous thrombosis | 124/265 | PLT <50 vs ≥100×10⁹/L | 0.51 (0.23–1.14) | 0.102 |
| Venous thrombosis | 124/265 | PLT 50–<100 vs ≥100×10⁹/L | 2.36 (1.16–4.81) | 0.018* |

Data are shown as adjusted odds ratios (aORs) with 95% confidence intervals. Patients with cardiac valve disease or microvascular disease were excluded, leaving 265 patients: 38 with PLT <50×10⁹/L, 51 with PLT 50–<100×10⁹/L, and 176 with PLT ≥100×10⁹/L. Models were adjusted for age, sex, study center, diabetes mellitus, smoking, APS subtype, and aGAPSS. PLT ≥100×10⁹/L was the reference group. **P*<0.05 was considered statistically significant.

**Supplementary Table S6. Baseline multivariable logistic regression replacing aGAPSS with triple-positive aPL profile**

| **Outcome (events/N)** | **PLT <50×10⁹/L** | **PLT 50–<100×10⁹/L** | **Triple-positive aPL** |
| --- | --- | --- | --- |
| Any thrombosis (224/300) | 0.76 (0.33–1.76), *P*=0.523 | 1.72 (0.72–4.13), *P*=0.225 | 0.40 (0.22–0.74), *P*=0.004* |
| Arterial thrombosis (130/300) | 2.31 (1.13–4.71), *P*=0.022* | 0.86 (0.44–1.67), *P*=0.653 | 0.78 (0.46–1.33), *P*=0.360 |
| Cerebral infarction (114/300) | 2.47 (1.23–4.97), *P*=0.011* | 0.89 (0.45–1.76), *P*=0.735 | 0.78 (0.46–1.35), *P*=0.379 |
| Venous thrombosis (138/300) | 0.76 (0.38–1.52), *P*=0.437 | 2.78 (1.40–5.52), *P*=0.003* | 0.47 (0.27–0.80), *P*=0.005* |

Data are shown as adjusted odds ratios (aORs) with 95% confidence intervals. Models were adjusted for age, sex, study center, hypertension, hyperlipidemia, diabetes mellitus, smoking, APS subtype, triple-positive aPL profile, and both platelet-count strata. PLT ≥100×10⁹/L was the reference group. The inverse associations observed for triple-positive aPL profile with any and venous thrombosis are cohort-specific and should not be interpreted as evidence of a protective effect. aGAPSS, adjusted Global Antiphospholipid Syndrome Score; aPL, antiphospholipid antibody; CI, confidence interval; PLT, platelet count. **P*<0.05.

**Supplementary Table S7. Cox sensitivity analyses additionally adjusted for triple-positive aPL profile**

| **Outcome** | **PLT <50×10⁹/L** | **PLT 50–<100×10⁹/L** | **Triple-positive aPL** |
| --- | --- | --- | --- |
| Any thrombotic event | 3.32 (1.56–7.08), *P*=0.002* | 1.96 (0.93–4.11), *P*=0.075 | 0.97 (0.51–1.85), *P*=0.932 |
| Arterial thrombotic event | 3.97 (1.40–11.23), *P*=0.009* | 0.78 (0.17–3.67), *P*=0.755 | 0.82 (0.30–2.23), *P*=0.695 |
| Cerebral infarction | 4.10 (1.44–11.66), *P*=0.008* | 0.79 (0.17–3.72), *P*=0.768 | 0.83 (0.31–2.27), *P*=0.720 |
| Venous-related thrombotic event | 1.19 (0.40–3.57), *P*=0.755 | 2.04 (0.90–4.64), *P*=0.088 | 0.91 (0.42–1.98), *P*=0.816 |

Data are shown as hazard ratios (HRs) with 95% confidence intervals. Cox models were adjusted for age, sex, APS subtype, triple-positive aPL profile, and both platelet-count strata; follow-up was truncated at 48 months. PLT ≥100×10⁹/L was the reference group. aPL, antiphospholipid antibody; APS, antiphospholipid syndrome; CI, confidence interval; HR, hazard ratio; PLT, platelet count. **P*<0.05.

**Supplementary Table S8. Extended Cox sensitivity analyses adjusted for baseline thrombotic history and treatment variables**

| **Outcome** | **Events in PLT <50×10⁹/L** | **Events in PLT 50–<100×10⁹/L** | **Events in PLT ≥100×10⁹/L** | **PLT <50×10⁹/L, extended sensitivity model** | **PLT 50–<100×10⁹/L, extended sensitivity model** |
| --- | --- | --- | --- | --- | --- |
| Any thrombotic event | 11/47 | 11/49 | 21/158 | 2.73 (1.21–6.15), *P*=0.016* | 1.85 (0.85–4.01), *P*=0.120 |
| Arterial thrombotic event | 7/47 | 2/49 | 9/158 | 3.90 (1.19–12.82), *P*=0.025* | 0.65 (0.14–3.13), *P*=0.592 |
| Cerebral infarction | 7/47 | 2/49 | 9/158 | 4.15 (1.26–13.65), *P*=0.019* | 0.64 (0.13–3.08), *P*=0.575 |
| Venous-related thrombotic event | 4/47 | 9/49 | 17/158 | 1.08 (0.34–3.44), *P*=0.901 | 1.77 (0.74–4.28), *P*=0.202 |

Data are shown as hazard ratios (HRs) with 95% CIs. The extended Cox sensitivity model was adjusted for age, sex, APS subtype, baseline arterial thrombosis, baseline venous thrombosis, antiplatelet therapy, anticoagulation, hydroxychloroquine, glucocorticoid use, and anti-CD20 therapy. PLT ≥100×10⁹/L was used as the reference group. Follow-up analyses were performed in the Renji follow-up cohort and truncated at 48 months. This table provides the PLT 50–<100×10⁹/L estimates to complement the main Cox analysis shown in Figure 3, which focuses on PLT <50×10⁹/L as the primary exposure of interest. Because of the limited number of outcome events, the extended models were considered sensitivity analyses and should be interpreted cautiously. **P*<0.05 was considered statistically significant.

**Supplementary Table S9. Incident and recurrent thrombotic events during 48-month follow-up according to platelet strata**

| **Outcome** | **Event type** | **PLT <50×10⁹/L** | **PLT 50–<100×10⁹/L** | **PLT ≥100×10⁹/L** |
| --- | --- | --- | --- | --- |
| Any thrombotic event | Incident | 2 | 1 | 8 |
|  | Recurrent | 9 | 10 | 13 |
|  | Total | 11 | 11 | 21 |
| Arterial thrombotic event | Incident | 1 | 1 | 7 |
|  | Recurrent | 6 | 1 | 2 |
|  | Total | 7 | 2 | 9 |
| Cerebral infarction | Incident | 1 | 1 | 8 |
|  | Recurrent | 6 | 1 | 1 |
|  | Total | 7 | 2 | 9 |
| Venous-related thrombotic event | Incident | 3 | 1 | 6 |
|  | Recurrent | 1 | 8 | 11 |
|  | Total | 4 | 9 | 17 |

Values are numbers of events. Incident events were defined as the first occurrence of the corresponding thrombotic category during follow-up in patients without that category at baseline. Recurrent events were defined as new events occurring during follow-up in patients with the corresponding thrombotic category at baseline. The total rows correspond to the combined event counts used in the primary survival analyses. Because category-specific risk-set denominators differed according to baseline history, percentages are not presented.

**Supplementary Table S10. Exploratory Cox regression analyses of recurrent any thrombosis according to platelet strata**

| **Model** | **Platelet stratum** | **HR** | **95% CI** | ***P*-value** |
| --- | --- | --- | --- | --- |
| Model 1: unadjusted | PLT <50×10⁹/L | 5.14 | 2.13–12.36 | <0.001* |
|  | PLT 50–<100×10⁹/L | 2.95 | 1.29–6.73 | 0.010* |
| Model 2: age and sex | PLT <50×10⁹/L | 5.40 | 2.22–13.15 | <0.001* |
|  | PLT 50–<100×10⁹/L | 3.07 | 1.34–7.03 | 0.008* |
| Model 3: age, sex, and APS subtype | PLT <50×10⁹/L | 5.15 | 2.11–12.60 | <0.001* |
|  | PLT 50–<100×10⁹/L | 3.09 | 1.34–7.14 | 0.008* |
| Model 4: Model 3 + aGAPSS | PLT <50×10⁹/L | 5.10 | 2.08–12.51 | <0.001* |
|  | PLT 50–<100×10⁹/L | 3.04 | 1.31–7.05 | 0.010* |
| Model 5: Model 3 + baseline thrombotic pattern | PLT <50×10⁹/L | 4.96 | 1.80–13.64 | 0.002* |
|  | PLT 50–<100×10⁹/L | 2.91 | 1.24–6.79 | 0.014* |

The analysis included only patients with a history of any thrombosis at baseline (N=213; 32 recurrent events). PLT ≥100×10⁹/L was the reference group. Model 5 adjusted for age, sex, APS subtype, and baseline arterial and venous thrombotic patterns. Because of the limited number of recurrent events, all models were considered exploratory and hypothesis-generating. APS, antiphospholipid syndrome; CI, confidence interval; HR, hazard ratio; PLT, platelet count. **P*<0.05 was considered statistically significant.

**Supplementary Table S11. Restricted analysis of incident arterial thrombotic events among patients without arterial thrombosis at baseline**

| **Platelet stratum** | **Patients without baseline arterial thrombosis, n** | **Incident arterial events, n (%)** | **Event times, months** |
| --- | --- | --- | --- |
| PLT <50×10⁹/L | 14 | 1 (7.1%) | 16 |
| PLT 50–<100×10⁹/L | 30 | 1 (3.3%) | 36 |
| PLT ≥100×10⁹/L | 96 | 7 (7.3%) | 1, 2, 4, 11, 11, 17, 37 |

The restricted analysis included 140 patients without arterial thrombosis at baseline. Only nine first-ever arterial events occurred during 48-month follow-up. Event-free survival did not differ across platelet strata (log-rank *P*=0.529), and crude event proportions were also not significantly different (Fisher–Freeman–Halton exact *P*=0.753). Because of the limited number of events, adjusted Cox modeling was not performed. PLT, platelet count.

**Supplementary Table S12. Cox sensitivity analyses stratified by APS subtype**

| **Cohort** | **Outcome** | **PLT <50×10⁹/L,**  **main model** | **PLT 50–<100×10⁹/L,**  **main model** | **PLT <50×10⁹/L,**  **reduced extended model** | **PLT 50–<100×10⁹/L, reduced extended model** |
| --- | --- | --- | --- | --- | --- |
| Primary APS | Any thrombotic event | 4.36 (1.47–12.95), *P*=0.008* | 2.52 (0.81–7.85), *P*=0.111 | 4.83 (1.37–17.12), *P*=0.015* | 3.18 (0.93–10.80), *P*=0.064 |
| Primary APS | Arterial thrombotic event | 11.23 (1.77–71.29), *P*=0.010* | 0.95 (0.09–9.69), *P*=0.969 | 17.05 (1.50–194.38), *P*=0.022* | 1.10 (0.10–12.66), *P*=0.940 |
| Primary APS | Cerebral infarction | 11.23 (1.77–71.29), *P*=0.010* | 0.95 (0.09–9.69), *P*=0.969 | 17.05 (1.50–194.38), *P*=0.022* | 1.10 (0.10–12.66), *P*=0.940 |
| Primary APS | Venous-related thrombotic event | 1.48 (0.30–7.41), *P*=0.631 | 3.14 (0.87–11.35), *P*=0.081 | 1.98 (0.32–12.17), *P*=0.463 | 3.02 (0.76–11.93), *P*=0.115 |
| SLE-APS | Any thrombotic event | 2.27 (0.76–6.76), *P*=0.141 | 1.86 (0.66–5.27), *P*=0.242 | 2.25 (0.75–6.73), *P*=0.147 | 2.06 (0.63–6.72), *P*=0.232 |
| SLE-APS | Arterial thrombotic event | 1.70 (0.40–7.13), *P*=0.469 | 0.41 (0.05–3.56), *P*=0.419 | 2.03 (0.45–9.08), *P*=0.354 | 0.35 (0.04–3.40), *P*=0.369 |
| SLE-APS | Cerebral infarction | 1.75 (0.41–7.52), *P*=0.451 | 0.45 (0.05–3.91), *P*=0.470 | 2.22 (0.49–10.12), *P*=0.304 | 0.38 (0.04–3.69), *P*=0.403 |
| SLE-APS | Venous-related thrombotic event | 1.15 (0.24–5.54), *P*=0.860 | 2.36 (0.74–7.49), *P*=0.145 | 1.20 (0.24–5.98), *P*=0.824 | 2.63 (0.67–10.31), *P*=0.166 |

Data are shown as HRs with 95% CIs. Main subgroup models were adjusted for age and sex. Reduced extended subgroup models were adjusted for age, sex, baseline arterial thrombosis, baseline venous thrombosis, antiplatelet therapy, anticoagulation, hydroxychloroquine, and glucocorticoid use. Anti-CD20 therapy was not included in the reduced extended subgroup models because of limited event numbers and the risk of model overfitting. PLT ≥100×10⁹/L was used as the reference group. Subgroup results should be interpreted cautiously because of limited event numbers and wide confidence intervals. **P*<0.05 was considered statistically significant. These subgroup analyses were exploratory and were not intended to establish definitive differences between APS subtypes.

**Supplementary Table S13. Baseline comparison between the Renji follow-up cohort and the Nantong baseline-only cohort**

| **Characteristic** | **Renji follow-up cohort (n=254)** | **Nantong baseline-only cohort (n=59)** | ***P*-value** |
| --- | --- | --- | --- |
| Age, years | 38 (30.2–54) | 32 (28–50) | 0.079 |
| Female, n (%) | 188 (74.0) | 53 (89.8) | 0.009* |
| Disease duration, months | 95.5 (38.8–153.8) | 24 (2–90) | <0.001* |
| BMI, kg/m² | 23.0 (20.4–26.1) | 23.2 (20.3–25.1) | 0.987 |
| PLT, ×10⁹/L | 139.5 (64.0–230.2) | 176.0 (111.5–233.0) | 0.079 |
| PLT <50×10⁹/L, n (%) | 47 (18.5) | 4 (6.8) | 0.028*† |
| PLT 50–<100×10⁹/L, n (%) | 49 (19.3) | 8 (13.6) |  |
| PLT ≥100×10⁹/L, n (%) | 158 (62.2) | 47 (79.7) |  |
| Primary APS, n (%) | 105 (41.3) | 24 (40.7) | 0.214‡ |
| SLE-associated APS, n (%) | 136 (53.5) | 35 (59.3) |  |
| Other APS subtype, n (%) | 13 (5.1) | 0 (0.0) |  |
| Hypertension, n (%) | 61 (24.0) | 10 (16.9) | 0.243 |
| Diabetes mellitus, n (%) | 18 (7.1) | 4 (6.8) | 1.000 |
| Hyperlipidemia, n (%) | 63 (24.8) | 7 (11.9) | 0.032* |
| aGAPSS | 9 (5–13) | 9 (4.5–13) | 0.938 |
| GAPSS | 11 (7–16) | 12 (7–16) | 0.910 |
| Any thrombosis at baseline, n (%) | 213 (83.9) | 24 (40.7) | <0.001* |
| Arterial thrombosis at baseline, n (%) | 114 (44.9) | 18 (30.5) | 0.044* |
| Cerebral infarction at baseline, n (%) | 100 (39.4) | 15 (25.4) | 0.045* |
| Venous thrombosis at baseline, n (%) | 139 (54.7) | 12 (20.3) | <0.001* |
| Lupus anticoagulant positive, n (%) | 211 (83.1) | 52 (88.1) | 0.339 |

Data are presented as median (interquartile range) or n (%). Continuous variables were compared using the Mann–Whitney *U* test; categorical variables were compared using the *χ²* test or exact tests, as appropriate. †*P* value refers to the overall platelet-stratum distribution; ‡*P* value refers to the overall APS-subtype distribution. Triple-positive aPL status was 36.2% at Renji and 47.5% at Nantong (*P*=0.110). Baseline antiplatelet therapy (19.7% vs 42.4%, *P*<0.001), anticoagulation (82.7% vs 59.3%, *P*<0.001), and anti-CD20 therapy (39.4% vs 16.9%, *P*=0.001) also differed between centers. **P*<0.05.

**Figure S1. Kaplan–Meier curves for 48-month event-free survival according to platelet strata in primary APS.** (A) Any thrombotic event; (B) arterial thrombotic event; (C) cerebral infarction; (D) venous-related thrombotic event. P values were calculated using the log-rank test. In the primary APS subgroup, all eight arterial thrombotic events were cerebral infarctions occurring in the same patients at identical event times; therefore, panels B and C overlap. These subgroup visualizations should be interpreted as exploratory because of the limited number of outcome events.

**Figure S2. Kaplan–Meier curves for 48-month event-free survival according to platelet strata in SLE-APS. (A) Any thrombotic event; (B) arterial thrombotic event; (C) cerebral infarction; (D) venous-related thrombotic event. *P* values were calculated using the log-rank test. These subgroup visualizations should be interpreted as exploratory because of the limited number of outcome events.**
